# DNA Methylation Associated With Diabetic Kidney Disease in Blood-Derived DNA

**DOI:** 10.3389/fcell.2020.561907

**Published:** 2020-10-15

**Authors:** Laura J. Smyth, Christopher C. Patterson, Elizabeth J. Swan, Alexander P. Maxwell, Amy Jayne McKnight

**Affiliations:** ^1^Centre for Public Health, Queen’s University Belfast, Belfast, United Kingdom; ^2^Regional Nephrology Unit, Belfast City Hospital, Belfast, United Kingdom

**Keywords:** epigenetic, diabetes, kidney, renal, methylation, association

## Abstract

A subset of individuals with type 1 diabetes will develop diabetic kidney disease (DKD). DKD is heritable and large-scale genome-wide association studies have begun to identify genetic factors that influence DKD. Complementary to genetic factors, we know that a person’s epigenetic profile is also altered with DKD. This study reports analysis of DNA methylation, a major epigenetic feature, evaluating methylome-wide loci for association with DKD. Unique features (*n* = 485,577; 482,421 CpG probes) were evaluated in blood-derived DNA from carefully phenotyped White European individuals diagnosed with type 1 diabetes with (cases) or without (controls) DKD (*n* = 677 samples). Explicitly, 150 cases were compared to 100 controls using the 450K array, with subsequent analysis using data previously generated for a further 96 cases and 96 controls on the 27K array, and *de novo* methylation data generated for replication in 139 cases and 96 controls. Following stringent quality control, raw data were quantile normalized and beta values calculated to reflect the methylation status at each site. The difference in methylation status was evaluated between cases and controls; resultant *P*-values for array-based data were adjusted for multiple testing. Genes with significantly increased (hypermethylated) and/or decreased (hypomethylated) levels of DNA methylation were considered for biological relevance by functional enrichment analysis using KEGG pathways. Twenty-two loci demonstrated statistically significant fold changes associated with DKD and additional support for these associated loci was sought using independent samples derived from patients recruited with similar inclusion criteria. Markers associated with *CCNL1* and *ZNF187* genes are supported as differentially regulated loci (*P* < 10^–8^), with evidence also presented for *AFF3*, which has been identified from a meta-analysis and subsequent replication of genome-wide association studies. Further supporting evidence for differential gene expression in CCNL1 and ZNF187 is presented from kidney biopsy and blood-derived RNA in people with and without kidney disease from NephroSeq. Evidence confirming that methylation sites influence the development of DKD may aid risk prediction tools and stimulate research to identify epigenomic therapies which might be clinically useful for this disease.

## Introduction

Diabetes and associated complications are major personal and public health concerns, with diabetic kidney disease (DKD) contributing a substantial financial burden to healthcare providers (Franciosi et al., 2013; Campbell et al., 2017; Arredondo et al., 2018; Disease et al., 2018; Kawaguchi et al., 2020). DKD develops in approximately one-third of individuals with diabetes and remains the most common primary diagnosis of chronic kidney disease (CKD) leading to end-stage kidney disease (ESKD) worldwide (Hill et al., 2014; US Renal and Data System., 2018; Lassalle et al., 2019; UK Renal Registry, 2019). Current treatments are based on modification of risk factors and include the reduction in elevated blood pressure, hyperglycemia and hyperlipidemia. Epidemiological evidence confirms that heritable factors play a major role in the development and progression of DKD, but despite the identification of several genetic loci associated with DKD most of the inherited risk factors remain unknown (Sandholm et al., 2012, 2013, 2014, 2017; Canadas-Garre et al., 2018, 2019; van Zuydam et al., 2018; Fu et al., 2019; Salem et al., 2019).

Emerging evidence for epigenetic phenomena has transformed investigations of heritable influences on disease and, complementary to genome-wide association studies (GWAS), it is now cost-effective to perform population-based studies of the epigenome (Rakyan et al., 2011b; Canadas-Garre et al., 2018; Kato and Natarajan, 2019; Kerr et al., 2020). Epigenetic modifications modulate gene expression without changing the DNA sequence; these may be either stably inherited or dynamic epigenetic marks. Methylation is a key epigenetic feature that plays an important role in chromosomal integrity and regulation of gene expression with different methylation profiles now being associated with many complex diseases (Murphy and Mill, 2014; Greenberg and Bourc’his, 2019; Bayoumi et al., 2020; Hoang et al., 2020). Epigenome-wide association studies (EWAS) have revealed methylation features associated with type 1 diabetes (Rakyan et al., 2011a; Stefan et al., 2014; Elboudwarej et al., 2016), gestational diabetes (Haertle et al., 2017; Dias et al., 2019; Howe et al., 2020), and type 2 diabetes (Florath et al., 2016; Walaszczyk et al., 2018; Ochoa-Rosales et al., 2020). EWAS have also identified methylation features associated with chronic kidney disease (Smyth et al., 2014a,b, 2018; Chu et al., 2017; Qiu et al., 2018).

Differential DNA methylation has been associated with “metabolic memory” of glycemic control and with a higher risk of developing DKD (Swan et al., 2015; Keating et al., 2018; Aranyi and Susztak, 2019; Gluck et al., 2019; Gu, 2019; Jia et al., 2019; Park et al., 2019). As DKD is not clinically detectable until significant organ damage has developed (albuminuria and/or reduced eGFR), more effective diagnostic tools and treatments, guided by a better understanding of pathophysiology, are urgently required. The identification of novel epigenetic risk markers and biological pathways influencing DKD would contribute more to the understanding of this serious diabetic complication. This manuscript describes an EWAS from carefully phenotyped individuals who were specifically recruited to investigate molecular risk factors for DKD in people with type 1 diabetes, followed by *in silico* and *de novo* replication. Our overarching aim is to identify differentially methylated CpG sites associated with DKD.

## Materials and Methods

### Participants

All recruited individuals provided written, informed consent and this study was approved by a United Kingdom Multicentre Research Ethics Committee (MREC/98/6/71). The discovery group comprised a subset of individuals (150 cases compared to 100 controls) selected from an established United Kingdom case-control collection that was recruited specifically to investigate risk factors for DKD (McKnight et al., 2010). One-third of the case group had end stage renal disease (ESRD). All individuals were White, from the United Kingdom and were diagnosed with type 1 diabetes prior to the age of 31 years. Participants in the case group had persistent proteinuria (>0.5 g protein/24 h) at least 10 years after diagnosis of diabetes, hypertension (BP > 135/85 mmHg or treatment with antihypertensive agents) and diabetic retinopathy. Individuals in the control group had at least 15 years duration of type 1 diabetes with normal renal function and were not receiving antihypertensive treatment. Cases and controls in the discovery group were matched for age, gender and duration of diabetes. The *in silico* replication groups comprised independent samples from the remainder of this collection and had similar characteristics to those involved in the discovery group. Similarly, all individuals included in the *de novo* typing phase were selected using blood-derived DNA from the larger collection (Table 1).

**TABLE 1:** Clinical characteristics of individuals participating in this study.

	**Discovery**		***In silico* replication**		***De novo* typing†**	
	**case**	**control**	**case**	**control**	**case**	**control**
N	150	100	96	96	139	96
Age at diagnosis (year)	15.8 ± 7.3	16.0 ± 7.1	16.2	16.3	16.86 ± 7.9	12.5 ± 7.3
Duration of diabetes (year)	26.6 ± 8.8	27.1 ± 8.6	27.5	27.8	30.1 ± 8.8	23.2 ± 7.4
HBA_1__c_ (%)	8.9 ± 2.0	8.8 ± 1.6	Not reported	Not reported	10.1 ± 2.0	9.5 ± 1.6
HBA_1__c_ (mmol/mol from www.ngsp.org/convert1.asp)	74	73			87	80
Male (%)	49	49	50.0	49.0	54.0	41.7

*†*De novo* genotyping group includes several individuals that were part of the *in silico* replication for cg02399570 and cg04861640 markers, which were not analyzed on the HumanMethylation27 BeadChip.*

### Methylation Typing

482,421 unique CpG features were evaluated in the discovery group. Existing blood-derived DNA [extracted using the salting-out method as previously described (Bell et al., 2010)] was accurately quantitated using PicoGreen^®^, normalized, and bisulfite treated using the EZ-96 DNA Methylation-Gold^TM^ Kit (Zymo Research, Irvine, CA, United States) with case and control samples randomly distributed across plates. The Infinium Human Methylation 450K BeadChip (Sandoval et al., 2011) (Illumina Inc., San Diego, CA, United States) was employed according to manufacturer’s instructions. Raw data were adjusted for dye bias and quantile normalized at the probe level with data derived from sites using Infinium I or Infinium II assay chemistry considered separately. This high-throughput platform enables quantitative evaluation of methylation levels with single nucleotide resolution, generating a methylation score per individual (a β value ranging from 0 for unmethylated to 1 representing complete methylation) for each CpG site.

*In silico* support for candidate loci was sought using normalized data available at the Gene Expression Omnibus accession GSE20067 (Bell et al., 2010). We previously analyzed 27,578 CpGs based on data from the Illumina Infinium^®^ HumanMethylation27 BeadChip in 192 individuals diagnosed as having type 1 diabetes with and without nephropathy using time to event (duration of diabetes until nephropathy) analysis (Bell et al., 2010).

*De novo* replication was performed using Sequenom Epityper assays (Sequenom Inc., Hamburg, Germany). Sequenom facilitates the quantitative analysis of DNA methylation using matrix-assisted laser desorption ionization time-of-flight mass spectrometry. Assays were designed using the default settings (except mass window range changed to 1500–8000) at www.epidesigner.com. Amplicons were carefully designed to cover target sites, results were generated following the manufacturer’s protocol and data analyzed using Epityper viewer 1.2 (Sequenom). An additional 235 independent individuals were typed as part of the replication population for *de novo* confirmation with the threshold for significance set at *P* < 0.05 without adjustment for multiple testing (Table 1).

### Analysis

For the discovery cohort, stringent quality control included evaluation of bisulfite conversion efficiency, staining, hybridization, target removal, extension, dye specificity and 600 integral negative controls. Samples were excluded where more than 10% of probes did not generate useful data and all sites with poor detection *P*-values (detection *P* ≥ 0.05) were set to “missing.” Known non-CpG targeting probes (*n* = 3,091) were excluded from all results (Chen et al., 2013; Zhou et al., 2017). Probes on autosomes were evaluated for association with DKD adjusted for sex, duration of diabetes, age at diagnosis, and HBA_1__c_ with subgroup analysis performed for cases with ESRD. Sex-specific analysis was performed for probes on chromosome X. Converting intensity levels to beta values and initial preprocessing were performed using the default settings within GenomeStudio’s methylation module v1.9 (Illumina) (Smyth et al., 2014b). Principal component analysis and multi-dimensional scaling were employed and potential outliers from gender, non-White ethnicity or experimental batch effects were excluded from further investigation. Proportional white cell counts from whole blood (B cells, granulocytes, monocytes, NK cells, and T cells subsets) were estimated using Houseman’s and Reinius’ approaches (Houseman et al., 2012; Reinius et al., 2012). Microarray quality control metrics reports were generated using the arrayQualityMetrics package with the recommended parameters in Bioconductor (Kauffmann and Huber, 2010); arrays that did not pass the default quality control thresholds were excluded from further analysis. After correction for dye bias, raw data were normalized using quantile normalization using methylumi^1^. The Bioconductor package Limma (Wettenhall and Smyth, 2004; Ritchie et al., 2015) was used to generate association results. Significance values for the 450K array-based analysis were adjusted for multiple testing using the Benjamini & Hochberg, “fdr” adjustment, implemented in limma and reported as adjusted *p*-values.

For *in silico* replication, previously generated association data from the Illumina Infinium^®^ HumanMethylation27 BeadChip for 192 individuals diagnosed as having type 1 diabetes with and without kidney disease (Bell et al., 2010) was “looked up.”

For *de novo* replication, new genotype data was generated using Sequenom’s MassARRAY^®^ System. Sequenom-based data were analyzed by the large samples *z* test statistic with regression analysis to adjust for sex, duration of diabetes, age at diagnosis, and HBA_1__*c.*_ The area under the receiver operating characteristic (ROC) curve was generated using SPSS (version 15) to assess the ability of a CpG to distinguish between cases and controls.

*In silico* functional support for replicated loci was sought from datasets within NephroSeq (last accessed 14th September 2020)^2^ for publicly available gene expression data using a *p*-value threshold of 0.05 and a fold change of at least 1.5.

To investigate if SNPs from our GWAS for DKD were located near differentially methylated CpG probes, SNPs in key chromosomal locations (5 kb flanking top ranked markers or their associated genes) were evaluated for association with DKD using publicly available data from our genome-wide association study (dbGAP phs000389.v1.p1); for SNP-based association analysis of this GWAS data, *P*-values were corrected by genomic control and adjusted for age at diagnosis, duration of diabetes, gender, biochemistry center and the first ten components of the study specific principal component analysis (Sandholm et al., 2012).

Provisionally significant genes (adjusted *P* < 0.0001) were analyzed for enrichment of KEGG pathway membership; enrichment was assessed for genes that showed increased or decreased methylation values separately. Additionally, DAVID Bioinformatics Resources 6.7 was interrogated for top-ranked gene results in the genetic association database, Online Mendelian Inheritance in Man, Kyoto Encyclopedia of Genes and Genomes (KEGG) pathways and articles recorded in PubMed (Huang da et al., 2009).

## Results

**FIGURE 1:**
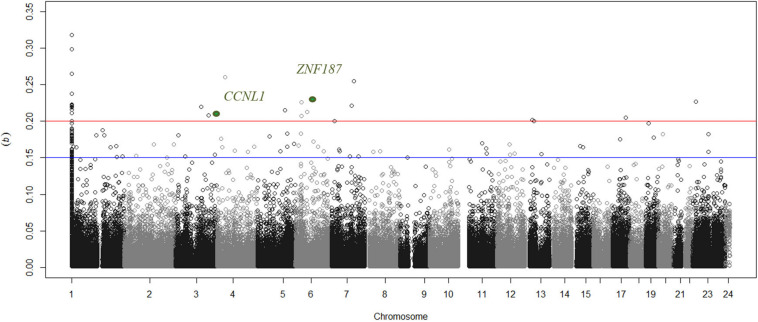
Manhattan plot showing distribution of delta beta values for uniquely mapped sites across all chromosomes. The blue line discriminates sites that have suggestive differences in methylation between cases with nephropathy compared to non-nephropathic individuals in the control group. Circles above the red line are loci where a substantial difference in beta values (>0.2) were observed and the two markers supported in the replication group are highlighted in green. Markers that are not uniquely mapped to a chromosome position based on Illumina’s updated bead pool manifest are assigned to chromosome 1 in this figure.

Greater than 99% concordance was observed between duplicate samples and experimentally defined genders matched each individual submitted for analysis based on Y-chromosome specific loci. Experimental controls generated expected results, but 5 arrays were identified as outliers and were thus removed from all subsequent analyses. There was no significant difference in the estimated proportional white cell counts between case and control groups (Supplementary Table 1). *In silico* adjustment for white cell composition does not alter the top-ranked genes associated with DKD in this study. As the discovery collection included 250 individuals, only CpGs with a sizable, significant difference in DNA methylation (Δβ) were considered for subsequent analysis (Figure 1). Twenty-two unique sites were identified with Δβ ≥ 0.2 and *P* < 10^–8^ where significance values were adjusted for multiple testing using the Benjamini & Hochberg method for controlling the false discovery rate (FDR). These sites are primarily “promoter associated” and affect 22 genes (Table 2); all 22 sites were taken forward for *in silico* and wet-lab replication. Subgroup analysis comparing those individuals who received a kidney transplant for DKD with controls show that 20 of these 22 sites were also highlighted in the ESRD focused dataset (Table 2). Sex-specific analyses for cg26399113 on the X chromosome was significant from female only (*P* = 1 × 10^–12^) and male only (*P* = 8.9 × 10^–11^) analyses. The discovery group generated results for 15 CpGs in the *CCNL1* gene, of which 12 showed an increase in methylation between cases and controls. Similarly for *ZNF187*, nine CpGs were examined of which eight showed differential methylation and all were in the same direction as the index marker.

**TABLE 2:** Details for markers showing significant (FDR adjusted *P*-value), substantial differences in mean methylation (Δβ) between case and control groups in the discovery phase.

**Locus**	**Sequence position**	**Cytogenetic location**	**Symbol**	**Role from Entrez Gene**	**Δβ DN vs. DC**	***P* DN vs. DC**	**Δβ ESRD vs. DC**	***P* ESRD vs. DC**
cg04861640	28234605	6p21.31	ZNF187 (ZSCAN26)	Transcriptional regulation	0.23	3.70E-24	0.22	3.80E-13
cg16203607	62500946	17q21	DDX5	Transcriptional regulation, differentiation, apoptosis	0.21	2.10E-23	0.21	1.90E-13
cg21829265	56911139	6p12.1	KIAA1586	Nucleic acid binding	0.21	4.10E-23	0.21	5.50E-13
cg20419181	38063874	4p14	TBC1D1	Regulation of cell growth, differentiation, protein organization	0.26	5.20E-23	0.27	9.00E-14
cg21935083	131892314	5q31	RAD50	Multiple including telomere maintenance and DNA repair/recombination	0.22	5.40E-23	0.23	3.10E-14
cg21739208	80280621	6	NA	NA	0.22	6.80E-23	0.22	8.10E-13
cg07242860	140739522	5q31	PCDHG@	Calcium ion binding	0.22	7.20E-23	0.23	1.20E-13
cg07979357	14142353	19p13.11	IL27RA	Immunity	0.2	1.80E-22	Not significant	Not significant
cg24115040	96652375	7q22	DLX5	Multiple, including transcriptional regulation, positive regulation of canonical Wnt receptor signaling pathway, and cell proliferation/differentiation	0.22	2.10E-22	0.23	4.70E-13
cg02339392	28234615	6p21.31	ZNF187 (ZSCAN26)	Transcriptional regulation	0.21	2.20E-22	0.21	7.90E-13
cg10463299	54987330	20q13.31	CASS4	Cell adhesion, cytoskeletal organization	0.23	2.30E-22	0.24	2.40E-13
cg00618312	41556559	13q13	ELF1	Protein acts as both an enhancer and a repressor to regulate transcription	0.2	2.40E-22	0.2	3.20E-12
cg15189015	72707255	17q25.1	RAB37;CD300LF	GTP binding; receptor activity	0.22	3.00E-22	0.23	6.40E-13
cg25418748	178977236	5q35.3	RUFY1	Endocytosis/protein transport	0.21	5.00E-22	0.21	1.20E-12
cg02399570	156877196	3q25.31	CCNL1	Transcription/RNA processing.	0.21	8.60E-22	0.21	3.30E-12
cg10905876	56716656	6p12.1	DST	Cell adhesion, cytoskeletal organization	0.22	8.90E-22	0.21	9.80E-12
cg16595484	122512170	3q21.1	HSPBAP1	Cell growth and differentiation	0.22	2.40E-21	0.22	1.30E-11
cg01667324	34392045	13q13.2	RFC3	Multiple including telomere maintenance and DNA repair/replication	0.2	5.30E-21	Not significant	Not significant
cg08779777	106505772	7q22.3	PIK3CG	Multiple. Associated with blood pressure variation (PMID: 21909110)	0.26	1.50E-20	0.25	6.10E-11
cg03037561	16459464	7p21.2	ISPD (CRPPA)	Nucleotidyltransferase activity, isoprenoid biosynthetic process	0.2	3.20E-18	0.24	1.50E-09
cg11671265	78722517	4q13.3	CNOT6L	Gene expression, RNA metabolic processing	0.24	5.00E-18	0.22	6.30E-09
cg26399113*	70752639	Xq13	OGT	Multiple, including response to insulin	0.22	1.00E-12	0.23	2.30E-07

Differential methylation between individuals with DKD (DN, *n* = 150) compared to controls (DC, *n* = 100) with diabetes but no evidence of renal disease are highlighted, alongside differential methylation and significance values for the smaller ESRD versus control dataset. * Results presented from female cases compared to female controls.

Functional enrichment analysis for differentially regulated loci (adjusted *P* < 0.0001) in the discovery group revealed both hypermethylation and hypomethylation across several pathways. Top ranked pathways enriched with significant probes include genes in Wnt signaling pathway (04310, enrichment *P* = 3.9 × 10^–5^), focal adhesion (04510, enrichment *P* = 7.9 × 10^–5^), MAPK signaling (04010, enrichment *P* = 1.0 × 10^–10^) pathways. Annotation from DAVID Bioinformatics Resources 6.7 for top-ranked genes is presented in Supplementary Table 2.

Several SNPs from our British - Irish genome-wide association study revealed nominal significance for SNPs in the region of interest from the 450K discovery analysis. The SNP demonstrating most evidence for association with DKD within 5 kb flanking top-ranked methylation probes are presented in Supplementary Table 3. Rs16888186 showed the most evidence for association in the *DST* gene with *P* = 0.0008.

**FIGURE 2:**
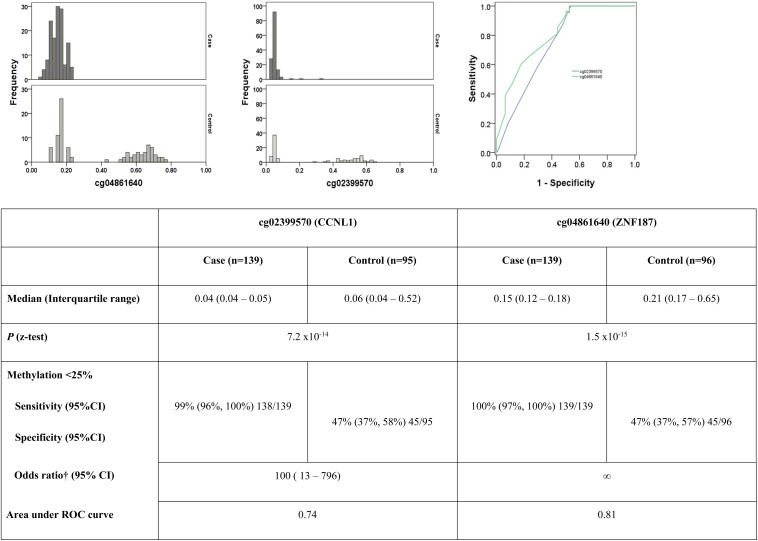
Histograms highlighting differences in the pattern of methylation between cases and controls for the two top ranked markers in the replication population. ROC curve demonstrating sensitivity and specificity of each marker. †Adjusted for age, HbA1c and gender. Neither age at diagnosis, nor duration of diabetes significantly influenced the model.

*In silico* replication data were available for six probes on the 27K array (cg25418748, cg07979357, cg21935083, cg21829265, cg24115040, cg26399113), but none of these sites confirmed differential methylation levels between cases and controls. *De novo* replication gave *z* test *P*-values supporting the association of two loci: cg02399570 on 3q25.31 in the *CCNL1* gene, *P* = 7.2 × 10^–14^ and cg04861640 on 6p21.31 in the *ZNF187* gene, *P* = 1.5 × 10^–15^ (Figure 2). Selecting a β level of 25% (as standard for unmethylated loci), the area under the ROC curve was 0.74 for cg02399570 and 0.81 for cg04861640 (Figure 2).

Multiple significant analyses for *CCNL1* gene expression associated with kidney function were returned from NephroSeq (Supplementary Figure 1). Higgins et al. (2004) observed that *CCNL1* is more highly expressed in renal glomeruli than other renal tissues. Downregulation of *CCNL1* gene expression was reported in multiple datasets comparing people with kidney disease versus controls without kidney disease [*P* = 3.55E-09 for thin basement membrane disease, based on 199 micro-dissected glomerular samples from CKD patients and living donors analyzed on Affymetrix Human U133 Plus 2.0 and Affymetrix Human U133A (altCDF v10) platforms, and *P* = 1.84E-05 for 53 kidney biopsies from people with chronic kidney disease tubular damage compared to healthy controls analyzed on Agilent Whole Human Genome Microarrays (Nakagawa et al., 2015)]. Gunther et al. (2014) reported a slight increase in blood-derived *CCNL1* gene expression (*P* = 0.006, fold change 1.6) for kidney transplant recipients with acute rejection compared to kidney transplant recipients with no rejection based on analysis of RNA from 40 samples on the Affymetrix Human Genome U133 Plus 2.0 array. Cox et al. (2015) explored blood-derived gene expression changes associated with glomerular filtration rate in eight patients with IgA nephropathy compared to nine healthy control participants, observing only a small change in expression for CCNL1 (*P* = 0.02).

Multiple significant analyses were returned from NephroSeq *ZNF187* kidney gene expression data from dissected renal lobes of five adult human kidneys using cDNA microarrays representing ∼30,000 different human genes (Higgins et al., 2004). ZNF187 is more highly expressed in renal glomeruli than other renal tissues (Higgins et al., 2004; Lindenmeyer et al., 2010). The most significant result returned from searching NephroSeq for ZNF187 was for tissue type [*P* = 1.7 × 10^–5^ comparing tubulointerstitium to glomerular tissue in six transplant living donors (Lindenmeyer et al., 2010)] and acute rejection following kidney transplantation from 48 patients [*P* = 2.8 × 10^–5^ (Sarwal et al., 2003)]. Gene expression changes were associated with glomerular filtration rate in kidney biopsy samples from people with IgA nephropathy (*P* = 6.27 × 10^–4^) (Reich et al., 2010), diabetes (*P* = 0.008) (Woroniecka et al., 2011), and blood derived gene expression from healthy individuals who had no evidence of kidney disease (*P* = 0.018) (Flechner et al., 2004). Focusing in on available data from 22 racially diverse microdissected human kidney samples with type 2 DKD measured on the Affymetrix U133A 2.0 array, visualizes decreased renal function associated with a decrease in ZNF187 gene expression (*P* = 0.008) (Woroniecka et al., 2011).

## Discussion

Using the Infinium Human Methylation 450K BeadChip (Sandoval et al., 2011) we identified differentially methylated CpG sites associated with DKD. To minimize false positive associations in our discovery cohort, we applied stringent quality control and adjusted association analyses; we used both a genome-wide significance threshold and a clear absolute methylation difference (Δβ ≥ 0.2) to minimize artifactual associations and ensure the selection of differentially methylated CpG probes (Dedeurwaerder et al., 2014). No SNPs are reported to affect methylation probes for *ZNF187*, however, one SNP resides 40 bases from the 3′ end of the probe for the *CCNL1* gene. This SNP (rs75624594, NP_064703.1:p.His112 = His, synonymous coding) has a reported minor allele frequency of 50% in African individuals, but only 4% in the US NHLBI Exome Sequencing Project (dbSNP ss342150967)^3^ so is unlikely to account for the differential methylation observed in this study.

The majority of probes evaluated using the 27K array were also present on the 450K array (*n* = 25,978), and demonstrate good correlation for many, but not all, CpG probes. For this reasons we sought supporting data from a previous study exploring DKD using the 27K array. It is clearly only possible to seek support for top-ranked markers that were present across both the 27K and 450K arrays and unfortunately data was only available for seven probes, none of which supported an association. Acknowledging the limitations of the 27K array to support top-ranked markers in this study (primarily that the majority of our top-ranked markers were not present on that much smaller array) and in the absence of a replication cohort with 450K data available for a population with similar phenotype characteristics, we sought independent replication. Pragmatically, independent replication was sought using all available samples with high molecular weight DNA (139 cases and 96 controls) for the 22 loci that were significantly associated with DKD from the 450K array-based discovery analysis using Δβ ≥ 0.2 and FDR adjusted *P* < 10^–8^ thresholds. Importantly, the replication population was recruited with similar phenotypic characteristics to that of the discovery cohort and we used a completely different wet-lab approach (mass spectrometry) to validate the microarray-based data, which minimizes artifacts due to the microarray analysis. Only two of these CpG sites were supported by the new methylation data generated in our replication population – this is not unusual in genome-wide studies and may be due to false positives in the original EWAS or due to the fact that our replication population was not sufficiently powered to identify significant associations with all loci. Both discovery and *de novo* replication using a different experimental platform (mass spectrometry by Sequenom analyzed using EpiTyper software) to generate new laboratory data and an independent population strongly support the association of *CCNL1* and *ZNF187* genes with methylome-wide significance from 450K array-based (microarray by Illumina’s iScan) discovery association results. RNA-based gene expression data also supports a functional influence of *CCNL1* and *ZNF187* in kidney disease.

Methylation patterns for *CCNL1* and *ZNF187* genes showed a striking higher methylation level for controls compared to individuals in the case group (Figure 2). It should be noted that DNA samples available for replication were not age and gender matched in this study, rather they were pragmatically selected as all available samples with high quality DNA and careful phenotyping. Cases were older, diagnosed with type 1 diabetes later, and had higher HBA_1__c_ values with a longer duration of diabetes than individuals in the control group. Differential methylation between cases and controls was significant in both the *CCNL1* and *ZNF187* genes following adjustment for age, duration of diabetes, HBA1c and gender.

The *CCNL1* gene encodes cyclin L1, which is localized in nuclear speckles (splicing factor storage compartment) (Herrmann et al., 2007), is functionally related to the spliceosome, and is involved in pre-mRNA splicing activities (Chen et al., 2007; Tannukit et al., 2008). The transcription start site for *CCNL1* is located 89 bp upstream of the initiation codon and the first two exons overlap the CpG island (Dickinson et al., 2002; Figure 2). *CCNL1* was consistently hypomethylated in cases compared to controls. Overexpression (usually associated with hypomethylation) of *CCNL1* has been associated with cancer (Sticht et al., 2005; Mitra et al., 2010; Peng et al., 2011). The discovery group generated results for 15 CpGs in the *CCNL1* gene, of which 12 showed an increase in methylation between cases and controls. Replication using independent samples and a different wet-lab experimental approach supported the association of *CCNL1* with DKD. Based on published gene expression data from the Affymetrix^®^ GeneChip^®^ Whole Transcript Expression Arrays, *CCNL1* was one of four genes differentially expressed in patients with kidney stones compared to controls (2.6 fold change, downregulated, *P* = 6.58E-05) (Liang et al., 2019). Based on gene expression data within NephroSeq, differential gene expression was observed for *CCNL1* in kidney biopsy tissues from people with kidney disease compared to controls (Gunther et al., 2014; Ju et al., 2015; Nakagawa et al., 2015); no adjustment was made for cell heterogeneity in the disease compared to control collections for these gene expression datasets. *CCNL1* resides in chromosome band 3q25, which has been previously suggested to harbor risk loci for DKD (McKnight et al., 2009). Of particular interest, the *CCNL1* gene was ranked 4th from a meta-analysis for association with severe diabetic retinopathy (*P* = 7.1 × 10^–7^), but was no longer top-ranked for association with diabetic retinopathy when individuals with nephropathy were removed from the case group (Grassi et al., 2011). Subsequent studies have highlighted *CCNL1* SNPs associated with retinopathy and measures of renal function (Lin et al., 2016). Genetic variation near the *CCNL1* gene is robustly associated with low birth weight in European individuals (Freathy et al., 2010; Yaghootkar and Freathy, 2012; Horikoshi et al., 2013), specifically with growth restriction from early pregnancy onward (Mook-Kanamori et al., 2011). Another study suggests that individuals who carry a risk allele for rs900400 (near *CCNL1*) are more vulnerable to stress impacting on birth weight (Ali Khan et al., 2012). The relationship between birth weight and kidney disease has been debated with some groups suggesting that low birth weight is a risk factor for DKD (Rossing et al., 1995) while others report that low birth weight does not increase the risk of DKD (Fagerudd et al., 2006). The birth weight lowering effect rs900400 C allele has also been associated with increased insulin response following oral glucose stimulation in a meta-analysis based on Danish and Finnish non-diabetic individuals (Andersson et al., 2011). Published literature suggests that *CCNL1* may affect an individual’s inherited and dynamic responses to their environment, perhaps reflecting both genetic and epigenetic contributions. While the molecular mechanism for *CCNL1* influencing DKD remains to be resolved, this is clearly a candidate gene that warrants further investigation having demonstrated genetic (SNPs), epigenetic (methylation) and transcriptomic (gene expression) associations with kidney disease across multiple collections.

In the discovery cohort, nine CpGs were examined for ZNF187; eight of these showed differential methylation and all were in the same direction as the index marker. Replication demonstrated strong support for association of ZNF187 with DKD. Analysis within NephroSeq revealed gene expression changes associated with glomerular filtration rate in kidney biopsy and blood-derived samples from people with IgA nephropathy (Reich et al., 2010), diabetes (Woroniecka et al., 2011), and renal function in healthy individuals (Flechner et al., 2004; Supplementary Figure 1). ZNF187 is involved with transcriptional regulation, but there are few publications describing this gene. The *ZNF187* gene is was located at 6p21.31 and encodes the zinc finger protein 187. Gene ontology suggests that *ZNF187* is involved with transcriptional regulation, but there are no specific publications for this gene (or any aliases) in PubMed (accessed 12/05/20)^4^. The protein coding *ZNF187* gene has been renamed as *ZSCAN26* (zinc finger and SCAN domain containing 26), but we have retained the *ZNF187* nomenclature throughout this manuscript to keep the methylation array information consistent and facilitate easier searching and replication within methylation and gene expression datasets.

Both CCNL1 and ZNF187 were more highly expressed in renal glomeruli than other renal tissues, which may be consistent with DKD in people with type 1 diabetes primarily affecting the glomerulus (Higgins et al., 2004). The results for both significantly replicated genes in our methylation dataset are hypomethylated in cases compared to controls, while publicly available gene expression evidence for both genes suggests there is less RNA product in samples from people with kidney disease. This may be because less gene is expressed, or because the mRNA measured on arrays is not present for long, or because different isoforms are not captured by the gene expression array. cg02399570 is in the body of CCNL1 while cg04861640 is within the transcription start site of ZNF187. Most promoters and CpG sites in gene bodies that are hypomethylated are basically expressed, but gene regulation is complex and genes may be hypomethylated and “overexpressed” in disease states (Haney et al., 2016; Li et al., 2017). Significant further research is required in populations that have blood derived, kidney biopsy derived and *in vitro* models with SNP, CpG, and gene expression data available for the same individuals to tease out the molecular signatures of these genes (*CCNL1* and *ZNF187*) for kidney disease.

Pathway analysis revealed significant gene enrichment in the focal adhesion, Wnt and MAPK signaling pathways. These pathways have been previously top-ranked as differentially regulated in renal tubuli of individuals with DKD compared to healthy tissue from living kidney donors (Woroniecka et al., 2011). The focal adhesion pathway also demonstrates enrichment in the glomeruli of individuals with DKD (Woroniecka et al., 2011) and is a key molecular pathway in the formation and progression of the cardiorenal system (Muhlberger et al., 2012). The Wnt signally pathway has been shown to influence survival of glomerular mesangial cells exposed to high glucose (Lin et al., 2006) (41) and dysregulation of the Wnt pathway may represent and important pathogenic mechanism of DKD (Kavanagh et al., 2011; Zhou et al., 2012).

We incorporated existing GWAS data (Sandholm et al., 2012) with this novel methylation data to provide exploratory analysis seeking a provisional assessment of functionality. i.e., are SNPs demonstrating suggestive association with DKD from GWAS near CpG probes that are differentially methylated using the same case-control study population. As SNPs may have a functional role for each gene at a considerable genetic distance, we pragmatically selected 5 kb flanking each probe for this analysis. No strong associations were identified for SNPs near top-ranked differentially methylated genes. We also considered a similar analysis by looking up CpG sites for association near top-ranked GWAS SNPs (Sandholm et al., 2012). Meta-analysis of genome-wide association studies for DKD revealed novel association (*P* = 1.2 × 10^–8^) with SNPs in the *AFF3* gene, a transcriptional activator that influences renal fibrosis through the TGFβ1 pathway, in individuals who had progressed to end stage renal disease (Sandholm et al., 2012). Additionally, the top-ranked marker associated with DKD was in the *ERBB4* gene (*P* = 2.1 × 10^–7^) (Sandholm et al., 2012). Significant differential methylation was observed at both of these loci when comparing cases and controls on the 450K array (Table 3) suggesting that a combined genetic-epigenetic factor may influence the risk of DKD.

**TABLE 3:** Methylation probes of interest associated with genes identified from recent meta-analysis of genome-wide association studies for DKD.

**Locus**	**Gene**	**Description**	**Δβ DN vs. DC**	***P* DN vs. DC**
cg15934776	AFF3	AF4/FMR2 family, member 3	−0.024	1.40E-13
cg13353679	AFF3	AF4/FMR2 family, member 3	−0.045	3.40E-12
cg23118464	AFF3	AF4/FMR2 family, member 3	−0.022	1.50E-10
cg11577355	AFF3	AF4/FMR2 family, member 3	0.019	3.00E-10
cg22039234	AFF3	AF4/FMR2 family, member 3	−0.017	4.60E-08
cg01286950	AFF3	AF4/FMR2 family, member 3	−0.01	1.70E-07
cg11451506	AFF3	AF4/FMR2 family, member 3	0.013	1.10E-06
cg03393607	AFF3	AF4/FMR2 family, member 3	0.0057	1.10E-06
cg03816062	AFF3	AF4/FMR2 family, member 3	0.0038	2.00E-06
cg25798409	ERBB4	v-erb-a erythroblastic leukemia viral oncogene homolog 4 (avian)	−0.025	4.80E-14
cg24768649	ERBB4	v-erb-a erythroblastic leukemia viral oncogene homolog 4 (avian)	−0.012	1.20E-06

The role of epigenetics in common, complex diseases is beginning to be unraveled at a population level using relatively high throughput tools. Evidence is increasing that inter-individual epigenetic variation, in particular DNA methylation, may help explain some of the “missing heritability” that has not been identified through genome-wide association and resequencing approaches. Illumina’s 450K BeadChip was proposed as the method of choice for cost-effective, high throughput epigenome-wide association studies with single-base resolution (Rakyan et al., 2011b). The content of this array (485,764 sites distributed across all chromosomes) was selected based on input from 22 methylation experts across the world. Included are unique markers that cover 99% of RefSeq genes with an average of 17 CpG sites per gene region distributed across the promoter, 5′ untranslated region, first exon, gene body, and 3′ untranslated region. This array also includes dedicated content for CpG sites outside CpG islands and microRNA promoter regions. Illumina have released a higher density EPIC array, the Infinium MethylationEPIC BeadChip, which facilitates evaluation of 862,927 sites at significantly increased financial cost. More comprehensive analysis of the methylome may be conducted through whole-methylome-sequencing, but this is financially prohibitive for most researchers using cohorts of more than 200 participants. However, technological and analytical advances now offer the potential for targeted, high throughput bisulfite sequencing with deep coverage as an attractive option for technical validation and replication. Using high density methylation arrays is currently the most cost-effective approach for EWAS using population-based study designs. Stringent quality control, strong significance values, and independent replication are essential to minimize false positive findings when investigating sequence changes to elucidate the genetic architecture of multifactorial disease.

Although this epigenetic study does not include the large sample numbers traditionally associated with genome-wide association studies, we have applied rigorous analysis approaches throughout and gained support using independent samples by a different technology – important to minimize technical artifacts. A sample size of only 65 heavy smokers and 56 non-smokers was sufficient to identify differential patterns of methylation (*P* = 2.68^–31^) associated with smoking using the Illumina Human Methylation 27K BeadChip (Breitling et al., 2011). Similarly, a type 1 diabetes-methylation variation position signature was detected by assaying a relatively modest number of samples (*n* = 15 monozygotic twin pairs discordant for type 1 diabetes) on the 27K array (Bell et al., 2010; Rakyan et al., 2011a). Associations using the 450K array have been reported from 11 cell lines for rheumatoid arthritis (Nakano et al., 2013), 48 individuals for irritable bowel disease (Harris et al., 2012), and 165 females for alcohol use (Philibert et al., 2012), although study sizes are now increasing. Previous studies exploring blood-derived DNA methylation used an EWAS approach for DKD focused on the 27K array (Sapienza et al., 2011), the mitochondrial genome (Swan et al., 2015) or renal function decline in 181 Pima Indians with diabetes (Qiu et al., 2018). Targeted DNA methylation studies have been conducted using blood-derived DNA (Aldemir et al., 2017; Smyth et al., 2018), cell models of DKD (Brennan et al., 2010; Li et al., 2019) and an EWAS reported on kidney biopsy samples from 91 individuals of whom 45% had diabetes (Gluck et al., 2019) and 11 individuals with diabetes (Ko et al., 2013). The case and control population employed in this study has >60% power to detect a true positive (defined as detected CpGs with a meaningful difference in mean blood derived DNA methylation ±0.2 with a false discovery rate *P* ≤ 0.05) association using pwrEWAS (Graw et al., 2019). More comprehensive whole genome bisulfite sequencing has been reported for kidney biopsy samples from five individuals with DKD compared to one person with diabetes without kidney disease, and four people with neither diabetes nor kidney disease (Park et al., 2019).

Careful phenotyping is critically important for methylation studies, as is the consistent extraction and storage of DNA. We and others have previously demonstrated that differences in DNA extraction approaches and storage methods significantly alter methylation profiles. Importantly for this study, all DNA was extracted using the same approach in the same laboratory (by two persons) with extracted DNA stored at −80°C in multiple aliquots with only one freeze-thaw cycle. Individuals were carefully phenotyped by consultant nephrologists using internationally agreed phenotype criteria. We restricted analysis to individuals with type 1 diabetes and known kidney function to minimize phenotypic heterogeneity and used a matched design for the discovery population.

Many cell types have unique methylation profiles so where possible it is important to adjust for cell heterogeneity in all studies using blood-derived or kidney-derived DNA. In our study, there was no significant difference in proportional white cell counts from whole blood (B cells, granulocytes, monocytes, NK cells, and T cells subsets) and adjusting for cell composition does not change the top-ranked association results for this study. While adjusting for white cell subpopulations is critically important for cancer studies, immune-mediated responses, and case-control approaches not matched for age and gender, this is a result that we and others have reported previously for carefully phenotyped populations with stringent wet-lab protocols from blood sampling through to array scanning. The proportion of these cell types may also reflect changing disease pathology (Lappalainen and Greally, 2017; Johnson et al., 2020). Epigenetic signatures may display tissue specificity linked to disease mechanisms, however, obtaining kidney biopsy material is invasive and is not performed as part of routine clinical practice in people with DKD and T1D in the United Kingdom. Peripheral blood-based methylation biomarkers have shown promise in several clinical fields (Moore et al., 2014; Agha et al., 2019; Cardenas et al., 2019; DiTroia et al., 2019; Henderson-Smith et al., 2019; Kerr et al., 2019a,b, 2020; Ladd-Acosta and Fallin, 2019; Zhou et al., 2019) including kidney disease (Smyth et al., 2014b, 2018; Swan et al., 2015; Aranyi and Susztak, 2019; Gluck et al., 2019; Kato and Natarajan, 2019; Kerr et al., 2019b; Park et al., 2019). We have previously demonstrated that blood-derived differential methylation is also reflected in kidney-derived differential methylation for CKD (Smyth et al., 2014b). Blood-derived DNA methylation offers clinical utility in biomarker development, incorporating a minimally invasive approach that could be cost-effectively implemented in a routine clinical setting. Indeed, ROC curve analysis suggests that 25% methylation for the two key *CCNL1* and *ZNF187* markers is reasonably good at differentiating individuals in case and control groups. This is particularly critical for translation in DKD given the difficulty obtaining serial renal biopsies to establish diagnosis, track progression, and monitor response (Kim et al., 2018). Array-based approaches using blood-derived DNA have previously identified risk factors and biomarkers associated with complex phenotypes (Sandholm et al., 2013, 2017; Keating et al., 2018; Canadas-Garre et al., 2019; Gu, 2019; Park et al., 2019).

Interpreting epigenetic factors as disease-causing or consequences of disease processes, alongside genetic and/or environmental heterogeneity, are a significant problem for complex disease. Nevertheless, this study demonstrates that using high density methylation arrays are an appropriate, cost-effective tool to identify differential methylation profiles that may deliver minimally invasive biomarkers that are relevant for diabetic complications. We have identified CCNL1 and ZNF187 as differentially methylated genes associated with DKD in multiple cohorts. Larger EWAS exploring more markers with larger sample sizes will deliver the same gains identifying molecular biomarkers as has been observed for GWAS in recent years. Using longitudinal cohort designs will allow researchers to observe how DNA methylation changes over time. More complex analytical tools are being developed for DNA methylation such as MethylNet (Levy et al., 2020), which offers further opportunities for novel discoveries and improved understanding. The integration of multi-omic profiling will lead to a better understanding of inherited susceptibility to DKD and biomarkers for this common disease.

## Data Availability Statement

The data supporting the conclusion of this article will be made available by the authors, without undue reservation.

## Ethics Statement

The studies involving human participants were reviewed and approved by the South and West Multicentre Research Ethics Committee (MREC/98/6/71). The patients/participants provided their written informed consent to participate in this study.

## Author Contributions

AMcK had full access to the data in the study and takes responsibility for the integrity of the data and the accuracy of the data analysis. LS, AM, and AMcK conceived and designed the study. LS and ES performed sample analysis. CP performed the statistical analyses in the replication group. All authors provided important intellectual content and agreed the final version of this manuscript.

## Conflict of Interest

The authors declare that the research was conducted in the absence of any commercial or financial relationships that could be construed as a potential conflict of interest.

## Footnotes

1 http://tinyurl.com/methylumi

2 www.nephroseq.org

3 https://esp.gs.washington.edu/drupal/

4 www.ncbi.nlm.nih.gov/pubmed

## References

[B1] Agha G. Mendelson M. M. Ward-Caviness C. K. Joehanes R. Huan T. Gondalia R. (2019). Blood leukocyte DNA methylation predicts risk of future myocardial infarction and coronary heart disease. *Circulation* 140 645–657. 31424985 10.1161/CIRCULATIONAHA.118.039357 PMC6812683

[B2] Aldemir O. Turgut F. Gokce C. (2017). The association between methylation levels of targeted genes and albuminuria in patients with early diabetic kidney disease. *Ren. Fail.* 39 597–601. 10.1080/0886022x.2017.1358180 28805547 PMC6446175

[B3] Ali Khan A. Rodriguez A. Sebert S. Kaakinen M. Cauchi S. Froguel P. (2012). The interplay of variants near LEKR and CCNL1 and social stress in relation to birth size. *PLoS One* 7:e38216. 10.1371/journal.pone.0038216 22685556 PMC3369922

[B4] Andersson E. A. Harder M. N. Pilgaard K. Pisinger C. Stancakova A. Kuusisto J. (2011). The birth weight lowering C-allele of rs900400 near LEKR1 and CCNL1 associates with elevated insulin release following an oral glucose challenge. *PLoS One* 6:e27096. 10.1371/journal.pone.0027096 22073261 PMC3208566

[B5] Aranyi T. Susztak K. (2019). Cytosine methylation studies in patients with diabetic kidney disease. *Curr. Diab. Rep.* 19:91. 10.1007/s11892-019-1214-6 31471761

[B6] Arredondo A. Orozco E. Duarte M. B. Cuadra M. Recaman A. L. Azar A. (2018). Trends and challenges in diabetes for middle-income countries: evidence from Mexico. *Glob. Public Health* 14 227–240. 10.1080/17441692.2018.1498115 30068257

[B7] Bayoumi A. Gronbaek H. George J. Eslam M. (2020). The epigenetic drug discovery landscape for metabolic-associated fatty liver disease. *Trends Genet.* 36 429–441. 10.1016/j.tig.2020.03.003 32396836

[B8] Bell C. G. Teschendorff A. E. Rakyan V. K. Maxwell A. P. Beck S. Savage D. A. (2010). Genome-wide DNA methylation analysis for diabetic nephropathy in type 1 diabetes mellitus. *BMC Med. Genom.* 3:33. 10.1186/1755-8794-3-33 20687937 PMC2924253

[B9] Breitling L. P. Yang R. Korn B. Burwinkel B. Brenner H. (2011). Tobacco-smoking-related differential DNA methylation: 27K discovery and replication. *Am. J. Hum. Genet.* 88 450–457. 10.1016/j.ajhg.2011.03.003 21457905 PMC3071918

[B10] Brennan E. P. Ehrich M. O’Donovan H. Brazil D. P. Crean J. K. Murphy M. (2010). DNA methylation profiling in cell models of diabetic nephropathy. *Epigenetics* 5 396–401. 10.4161/epi.5.5.12077 20458172

[B11] Campbell J. A. Bishu K. G. Walker R. J. Egede L. E. (2017). Trends of medical expenditures and quality of life in US adults with diabetes: the medical expenditure panel survey, 2002-2011. *Health Qual. Life Outcomes* 15:70. 10.1186/s12955-017-0651-7 PMC5390377 28407776

[B12] Canadas-Garre M. Anderson K. Cappa R. Skelly R. Smyth L. J. McKnight A. J. (2019). Genetic susceptibility to chronic kidney disease - some more pieces for the heritability puzzle. *Front. Genet.* 10:453. 10.3389/fgene.2019.00453 31214239 PMC6554557

[B13] Canadas-Garre M. Anderson K. McGoldrick J. Maxwell A. P. McKnight A. J. (2018). Genomic approaches in the search for molecular biomarkers in chronic kidney disease. *J. Transl. Med.* 16:292. 10.1186/s12967-018-1664-7 PMC6203198 30359254

[B14] Cardenas A. Lutz S. M. Everson T. M. Perron P. Bouchard L. Hivert M. F. (2019). Placental DNA methylation mediates the association of prenatal maternal smoking on birth weight. *Am. J. Epidemiol.* 188 1878–1886. 10.1093/aje/kwz184 31497855 PMC6825837

[B15] Chen H. H. Wong Y. H. Geneviere A. M. Fann M. J. (2007). CDK13/CDC2L5 interacts with L-type cyclins and regulates alternative splicing. *Biochem. Biophys. Res. Commun.* 354 735–740. 10.1016/j.bbrc.2007.01.049 17261272

[B16] Chen Y. A. Lemire M. Choufani S. Butcher D. T. Grafodatskaya D. Zanke B. W. (2013). Discovery of cross-reactive probes and polymorphic CpGs in the Illumina Infinium HumanMethylation450 microarray. *Epigenetics* 8 203–209. 10.4161/epi.23470 23314698 PMC3592906

[B17] Chu A. Y. Tin A. Schlosser P. Ko Y. A. Qiu C. Yao C. (2017). Epigenome-wide association studies identify DNA methylation associated with kidney function. *Nat. Commun.* 8:1286. 10.1038/s41467-017-01297-7 PMC5668367 29097680

[B18] Cox S. N. Serino G. Sallustio F. Blasi A. Rossini M. Pesce F. (2015). Altered monocyte expression and expansion of non-classical monocyte subset in IgA nephropathy patients. *Nephrol. Dial. Transplant.* 30 1122–1232. 10.1093/ndt/gfv017 25770168

[B19] Dedeurwaerder S. Defrance M. Bizet M. Calonne E. Bontempi G. Fuks F. (2014). A comprehensive overview of infinium humanmethylation450 data processing. *Brief Bioinform.* 15 929–941. 10.1093/bib/bbt054 23990268 PMC4239800

[B20] Dias S. Adam S. Rheeder P. Louw J. Pheiffer C. (2019). Altered genome-wide DNA methylation in peripheral blood of south african women with gestational diabetes mellitus. *Int. J. Mol. Sci.* 20:5828. 10.3390/ijms20235828 31757015 PMC6928622

[B21] Dickinson L. A. Edgar A. J. Ehley J. Gottesfeld J. M. (2002). Cyclin L is an RS domain protein involved in pre-mRNA splicing. *J. Biol. Chem.* 277 25465–25473. 10.1074/jbc.m202266200 11980906

[B22] Disease G. B. D. Injury I. Prevalence C. (2018). Global, regional, and national incidence, prevalence, and years lived with disability for 354 diseases and injuries for 195 countries and territories, 1990-2017: a systematic analysis for the Global Burden of Disease Study 2017. *Lancet* 392 1789–1858. 30496104 10.1016/S0140-6736(18)32279-7 PMC6227754

[B23] DiTroia S. P. Percharde M. Guerquin M. J. Wall E. Collignon E. Ebata K. T. (2019). Maternal vitamin C regulates reprogramming of DNA methylation and germline development. *Nature* 573 271–275. 10.1038/s41586-019-1536-1 31485074 PMC8423347

[B24] Elboudwarej E. Cole M. Briggs F. B. Fouts A. Fain R. Quach H. (2016). Hypomethylation within gene promoter regions and type 1 diabetes in discordant monozygotic twins. *J. Autoimmun.* 68 23–29. 10.1016/j.jaut.2015.12.003 26782299 PMC4792657

[B25] Fagerudd J. Forsblom C. Pettersson-Fernholm K. Saraheimo M. Waden J. Ronnback M. (2006). Low birth weight does not increase the risk of nephropathy in Finnish type 1 diabetic patients. *Nephrol. Dial. Transplant.* 21 2159–2165. 10.1093/ndt/gfl217 16702205

[B26] Flechner S. M. Kurian S. M. Head S. R. Sharp S. M. Whisenant T. C. Zhang J. (2004). Kidney transplant rejection and tissue injury by gene profiling of biopsies and peripheral blood lymphocytes. *Am. J. Transplant.* 4 1475–1489. 10.1111/j.1600-6143.2004.00526.x 15307835 PMC2041877

[B27] Florath I. Butterbach K. Heiss J. Bewerunge-Hudler M. Zhang Y. Schottker B. (2016). Type 2 diabetes and leucocyte DNA methylation: an epigenome-wide association study in over 1,500 older adults. *Diabetologia* 59 130–138. 10.1007/s00125-015-3773-7 26433941

[B28] Franciosi M. Lucisano G. Amoretti R. Capani F. Bruttomesso D. Di Bartolo. (2013). Nicolucci, A., Costs of treatment and complications of adult type 1 diabetes. *Nutr. Metab Cardiovasc. Dis.* 23 606–611. 10.1016/j.numecd.2012.03.002 22749531

[B29] Freathy R. M. Mook-Kanamori D. O. Sovio U. Prokopenko I. Timpson N. J. Berry D. J. (2010). Variants in ADCY5 and near CCNL1 are associated with fetal growth and birth weight. *Nat. Genet.* 42 430–435. 20372150 10.1038/ng.567 PMC2862164

[B30] Fu H. Liu S. Bastacky S. I. Wang X. Tian X. J. Zhou D. (2019). Diabetic kidney diseases revisited: a new perspective for a new era. *Mol. Metab* 30 250–263. 10.1016/j.molmet.2019.10.005 31767176 PMC6838932

[B31] Gluck C. Qiu C. Han S. Y. Palmer M. Park J. Ko Y. A. (2019). Kidney cytosine methylation changes improve renal function decline estimation in patients with diabetic kidney disease. *Nat. Commun.* 10:2461. 10.1038/s41467-019-10378-8 PMC6549146 31165727

[B32] Grassi M. A. Tikhomirov A. Ramalingam S. Below J. E. Cox N. J. Nicolae D. L. (2011). Genome-wide meta-analysis for severe diabetic retinopathy. *Hum. Mol. Genet.* 20 2472–2481. 10.1093/hmg/ddr121 21441570 PMC3098732

[B33] Graw S. Henn R. Thompson J. A. Koestler D. C. (2019). pwrEWAS: a user-friendly tool for comprehensive power estimation for epigenome wide association studies (EWAS). *BMC Bioinform.* 20:218. 10.1186/s12859-019-2804-7 31035919 PMC6489300

[B34] Greenberg M. V. C. Bourc’his D. (2019). The diverse roles of DNA methylation in mammalian development and disease. *Nat. Rev. Mol. Cell Biol.* 20 590–607. 10.1038/s41580-019-0159-6 31399642

[B35] Gu H. F. (2019). Genetic and epigenetic studies in diabetic kidney disease. *Front. Genet.* 10:507. 10.3389/fgene.2019.00507 31231424 PMC6566106

[B36] Gunther O. P. Shin H. Ng R. T. McMaster W. R. McManus B. M. Keown A. (2014). Novel multivariate methods for integration of genomics and proteomics data: applications in a kidney transplant rejection study. *OMICS* 18 682–695. 10.1089/omi.2014.0062 25387159 PMC4229708

[B37] Haertle L. El Hajj N. Dittrich M. Muller T. Nanda I. Lehnen H. (2017). Epigenetic signatures of gestational diabetes mellitus on cord blood methylation. *Clin. Epigenet.* 9:28. 10.1186/s13148-017-0329-3 PMC5368916 28360945

[B38] Haney S. L. Upchurch G. M. Opavska J. Klinkebiel D. Hlady R. A. Suresh A. (2016). Promoter hypomethylation and expression is conserved in mouse chronic lymphocytic leukemia induced by decreased or inactivated Dnmt3a. *Cell Rep.* 15 1190–1201. 10.1016/j.celrep.2016.04.004 27134162 PMC4864108

[B39] Harris R. A. Nagy-Szakal D. Pedersen N. Opekun A. Bronsky J. Munkholm P. (2012). Genome-wide peripheral blood leukocyte DNA methylation microarrays identified a single association with inflammatory bowel diseases. *Inflamm. Bowel. Dis.* 18 2334–2341. 10.1002/ibd.22956 22467598 PMC3812910

[B40] Henderson-Smith A. Fisch K. M. Hua J. Liu G. Ricciardelli E. Jepsen K. (2019). DNA methylation changes associated with Parkinson’s disease progression: outcomes from the first longitudinal genome-wide methylation analysis in blood. *Epigenetics* 14 365–382. 10.1080/15592294.2019.1588682 30871403 PMC6557551

[B41] Herrmann A. Fleischer K. Czajkowska H. Muller-Newen G. Becker W. (2007). Characterization of cyclin L1 as an immobile component of the splicing factor compartment. *FASEB J.* 21 3142–3152. 10.1096/fj.07-8377com 17494991

[B42] Higgins J. P. Wang L. Kambham N. Montgomery K. Mason V. Vogelmann S. U. (2004). Gene expression in the normal adult human kidney assessed by complementary DNA microarray. *Mol. Biol. Cell* 15 649–656. 10.1091/mbc.e03-06-0432 14657249 PMC329285

[B43] Hill C. J. Cardwell C. R. Patterson C. C. Maxwell A. P. Magee G. M. Young R. J. (2014). Chronic kidney disease and diabetes in the national health service: a cross-sectional survey of the U.K. national diabetes audit. *Diabet. Med.* 31 448–454. 10.1111/dme.12312 24102856

[B44] Hoang T. T. Sikdar S. Xu C. J. Lee M. K. Cardwell J. Forno E. (2020). Epigenome-Wide association study of DNA methylation and adult asthma in the agricultural lung health study. *Eur. Respir. J.* 56:2000217. 10.1183/13993003.00217-2020 PMC7469973 32381493

[B45] Horikoshi M. Yaghootkar H. Mook-Kanamori D. O. Sovio U. Taal H. R. Hennig B. J. (2013). New loci associated with birth weight identify genetic links between intrauterine growth and adult height and metabolism. *Nat. Genet.* 45 76–82. 23202124 10.1038/ng.2477 PMC3605762

[B46] Houseman E. A. Accomando W. P. Koestler D. C. Christensen B. C. Marsit C. J. Nelson H. H. (2012). DNA methylation arrays as surrogate measures of cell mixture distribution. *BMC Bioinform.* 13:86. 10.1186/1471-2105-13-86 22568884 PMC3532182

[B47] Howe C. G. Cox B. Fore R. Jungius J. Kvist T. Lent S. (2020). Maternal gestational diabetes mellitus and newborn DNA methylation: findings from the pregnancy and childhood epigenetics consortium. *Diabetes Care* 43 98–105. 31601636 10.2337/dc19-0524 PMC6925578

[B48] Huang, da W. Sherman B. T. Lempicki R. A. (2009). Systematic and integrative analysis of large gene lists using DAVID bioinformatics resources. *Nat. Protoc.* 4 44–57. 10.1038/nprot.2008.211 19131956

[B49] Jia Y. Reddy M. A. Das S. Oh H. J. Abdollahi M. Yuan H. (2019). Dysregulation of histone H3 lysine 27 trimethylation in transforming growth factor-beta1-induced gene expression in mesangial cells and diabetic kidney. *J. Biol. Chem.* 294 12695–12707. 10.1074/jbc.ra119.007575 31266808 PMC6709639

[B50] Johnson R. K. Vanderlinden L. A. Dong F. Carry M. Seifert J. Waugh K. (2020). Longitudinal DNA methylation differences precede type 1 diabetes. *Sci. Rep.* 10:3721. 10.1038/s41598-020-60758-0 PMC7048736 32111940

[B51] Ju W. Nair V. Smith S. Zhu L. Shedden K. Song P. (2015). Tissue transcriptome-driven identification of epidermal growth factor as a chronic kidney disease biomarker. *Sci. Transl. Med.* 7:316ra193. 10.1126/scitranslmed.aac7071 PMC4861144 26631632

[B52] Kato M. Natarajan R. (2019). Epigenetics and epigenomics in diabetic kidney disease and metabolic memory. *Nat. Rev. Nephrol.* 15 327–345. 10.1038/s41581-019-0135-6 30894700 PMC6889804

[B53] Kauffmann A. Huber W. (2010). Microarray data quality control improves the detection of differentially expressed genes. *Genomics* 95 138–142. 10.1016/j.ygeno.2010.01.003 20079422

[B54] Kavanagh D. H. Savage D. A. Patterson C. C. McKnight A. J. Crean J. K. Maxwell A. P. (2011). Warren 3 Uk GoKin. association analysis of canonical wnt signalling genes in diabetic nephropathy. *PLoS One* 6:e23904. 10.1371/journal.pone.0023904 21876774 PMC3158097

[B55] Kawaguchi H. Moriyama M. Hashimoto H. (2020). Does disease management for diabetic nephropathy reduce medical expenditure? evidence from a three-period difference-in-differences analysis. *BMC Health Serv. Res.* 20:403. 10.1186/s12913-020-05297-0 32393380 PMC7212603

[B56] Keating S. T. van Diepen J. A. Riksen N. P. El-Osta A. (2018). Epigenetics in diabetic nephropathy, immunity and metabolism. *Diabetologia* 61 6–20. 10.1007/s00125-017-4490-1 29128937 PMC6448927

[B57] Kerr K. McAneney A. Smyth L. Flanagan C. Silvestri J. Nesbitt M. A. (2019a). A systematic review of differential methylation in rare ophthalmic diseases. *BMJ Open Opthalmol.* 4:e000342. 10.1136/bmjophth-2019-000342 31799411 PMC6861117

[B58] Kerr K. McAneney H. Flanagan C. Maxwell A. P. McKnight A. J. (2019b). Differential methylation as a diagnostic biomarker of rare renal diseases: a systematic review. *BMC Nephrol.* 20:320. 10.1186/s12882-019-1517-5 31419951 PMC6697952

[B59] Kerr K. McAneney H. Smyth L. J. Bailie C. McKee S. McKnight A. J. (2020). A scoping review and proposed workflow for multi-omic rare disease research. *Orphanet. J. Rare Dis.* 15:107. 10.1186/s13023-020-01376-x PMC7189570 32345347

[B60] Kim H. Wang X. Jin (2018). Developing DNA methylation-based diagnostic biomarkers. *J. Genet. Genom.* 45 87–97. 10.1016/j.jgg.2018.02.003 29496486 PMC5857251

[B61] Ko Y. A. Mohtat D. Suzuki M. Park A. S. Izquierdo M. C. Han S. Y. (2013). Cytosine methylation changes in enhancer regions of core pro-fibrotic genes characterize kidney fibrosis development. *Genome Biol.* 14:R108. 10.1186/gb-2013-14-10-r108 PMC4053753 24098934

[B62] Ladd-Acosta C. Fallin M. D. (2019). DNA methylation signatures as biomarkers of prior environmental exposures. *Curr. Epidemiol. Rep.* 6 1–13. 10.1007/s40471-019-0178-z 31032172 PMC6481677

[B63] Lappalainen T. Greally J. M. (2017). Associating cellular epigenetic models with human phenotypes. *Nat. Rev. Genet.* 18 441–451. 10.1038/nrg.2017.32 28555657

[B64] Lassalle M. Monnet E. Ayav C. Hogan J. Moranne O. Couchoud C. (2019). 2017 Annual report digest of the Renal Epidemiology Information Network (REIN) registry. *Transpl. Int.* 32 892–902. 10.1111/tri.13466 31148236

[B65] Levy J. J. Titus A. J. Petersen C. L. Chen Y. Salas L. A. Christensen B. C. (2020). MethylNet: an automated and modular deep learning approach for DNA methylation analysis. *BMC Bioinform.* 21:108. 10.1186/s12859-020-3443-8 32183722 PMC7076991

[B66] Li W. Sargsyan D. Wu R. Li S. Wang L. Cheng D. (2019). DNA Methylome and transcriptome alterations in high glucose-induced diabetic nephropathy cellular model and identification of novel targets for treatment by tanshinone IIA. *Chem. Res. Toxicol.* 32 1977–1988. 10.1021/acs.chemrestox.9b00117 31525975 PMC8182679

[B67] Li Z. Zhuang X. Zeng J. Tzeng C. M. (2017). Integrated analysis of DNA methylation and mrna expression profiles to identify key genes in severe oligozoospermia. *Front. Physiol.* 8:261. 10.3389/fphys.2017.00261 28553232 PMC5427114

[B68] Liang X. Lai Y. Wu W. Chen D. Zhong F. Huang J. (2019). LncRNA-miRNA-mRNA expression variation profile in the urine of calcium oxalate stone patients. *BMC Med. Genom.* 12:57. 10.1186/s12920-019-0502-y 31036010 PMC6489260

[B69] Lin C. L. Wang J. Y. Huang Y. T. Kuo Y. H. Surendran K. Wang F. S. (2006). Wnt/beta-catenin signaling modulates survival of high glucose-stressed mesangial cells. *J. Am. Soc. Nephrol.* 17 2812–2820. 10.1681/asn.2005121355 16943306

[B70] Lin X. Wang J. Yun L. Jiang S. Li L. Chen X. (2016). Association between LEKR1-CCNL1 and IGSF21-KLHDC7A gene polymorphisms and diabetic retinopathy of type 2 diabetes mellitus in the Chinese Han population. *J. Gene Med.* 18 282–287. 10.1002/jgm.2926 27607899

[B71] Lindenmeyer M. T. Eichinger F. Sen K. Anders H. J. Edenhofer I. Mattinzoli D. (2010). Systematic analysis of a novel human renal glomerulus-enriched gene expression dataset. *PLoS One* 5:e11545. 10.1371/journal.pone.0011545 20634963 PMC2902524

[B72] McKnight A. J. O’Donoghue D. Maxwell A. (2009). Annotated chromosome maps for renal disease. *Hum. Mutat.* 30 314–320. 10.1002/humu.20885 19085929

[B73] McKnight A. J. Patterson C. C. Pettigrew K. A. Savage D. A. Kilner J. Murphy M. (2010). A GREM1 gene variant associates with diabetic nephropathy. *J. Am. Soc. Nephrol.* 21 773–781. 10.1681/asn.2009070773 20150533 PMC2865734

[B74] Mitra S. Mazumder Indra D. Basu S. Mondal R. K. Roy A. Roychoudhury S. (2010). Amplification of CyclinL1 in uterine cervical carcinoma has prognostic implications. *Mol. Carcinog* 49 935–943. 10.1002/mc.20671 20721974

[B75] Mook-Kanamori D. O. Marsh J. A. Warrington N. M. Taal H. R. Newnham J. P. Beilin L. J. (2011). Variants near CCNL1/LEKR1 and in ADCY5 and fetal growth characteristics in different trimesters. *J. Clin. Endocrinol. Metab* 96 E810–E815. 21307140 10.1210/jc.2010-2316

[B76] Moore K. McKnight A. J. Craig D. O’Neill F. (2014). Epigenome-wide association study for Parkinson’s disease. *Neuromol. Med.* 16 845–855. 10.1007/s12017-014-8332-8 25304910

[B77] Muhlberger I. Monks K. Fechete R. Mayer G. Oberbauer R. Mayer B. (2012). Molecular pathways and crosstalk characterizing the cardiorenal syndrome. *OMICS* 16 105–112. 10.1089/omi.2011.0121 22401656

[B78] Murphy T. M. Mill J. (2014). Epigenetics in health and disease: heralding the EWAS era. *Lancet* 383 1952–1954. 10.1016/s0140-6736(14)60269-5 24630775

[B79] Nakagawa S. Nishihara K. Miyata H. Shinke H. Tomita E. Kajiwara M. (2015). Molecular markers of tubulointerstitial fibrosis and tubular cell damage in patients with chronic kidney disease. *PLoS One* 10:e0136994. 10.1371/journal.pone.0136994 26317775 PMC4552842

[B80] Nakano K. Whitaker J. W. Boyle D. L. Wang W. Firestein G. S. (2013). DNA methylome signature in rheumatoid arthritis. *Ann. Rheum. Dis.* 72 110–117. 10.1136/annrheumdis-2012-201526 22736089 PMC3549371

[B81] Ochoa-Rosales C. Portilla-Fernandez E. Nano J. Wilson R. Lehne B. Mishra P. (2020). Epigenetic link between statin therapy and Type 2 diabetes. *Diabetes Care* 43 875–884. 32033992 10.2337/dc19-1828

[B82] Park J. Guan Y. Sheng X. Gluck C. Seasock M. J. Hakimi A. A. (2019). Functional methylome analysis of human diabetic kidney disease. *JCI Insight* 4:e128886. 10.1172/jci.insight.128886 PMC6629092 31167971

[B83] Peng L. Yanjiao M. Ai-guo W. Pengtao G. Jianhua L. Ju Y. (2011). A fine balance between CCNL1 and TIMP1 contributes to the development of breast cancer cells. *Biochem. Biophys. Res. Commun.* 409 344–349. 10.1016/j.bbrc.2011.05.021 21586274

[B84] Philibert R. A. Plume J. M. Gibbons F. X. Brody G. H. Beach S. R. (2012). The impact of recent alcohol use on genome wide DNA methylation signatures. *Front. Genet.* 3:54. 10.3389/fgene.2012.00054 22514556 PMC3322340

[B85] Qiu C. Hanson R. L. Fufaa G. Kobes S. Gluck C. Huang J. (2018). Cytosine methylation predicts renal function decline in American Indians. *Kidney Int.* 93 1417–1431. 10.1016/j.kint.2018.01.036 29709239 PMC5973533

[B86] Rakyan V. K. Beyan H. Down T. A. Hawa M. I. Maslau S. Aden D. (2011a). Identification of type 1 diabetes-associated DNA methylation variable positions that precede disease diagnosis. *PLoS Genet.* 7:e1002300. 10.1371/journal.pgen.1002300 21980303 PMC3183089

[B87] Rakyan V. K. Down T. A. Balding D. J. Beck S. (2011b). Epigenome-wide association studies for common human diseases. *Nat. Rev. Genet.* 12 529–541. 10.1038/nrg3000 21747404 PMC3508712

[B88] Reich H. N. Tritchler D. Cattran D. C. Herzenberg A. M. Eichinger F. Boucherot A. (2010). A molecular signature of proteinuria in glomerulonephritis. *PLoS One* 5:e13451. 10.1371/journal.pone.0013451 20976140 PMC2956647

[B89] Reinius L. E. Acevedo N. Joerink M. Pershagen G. Dahlen S. E. Greco D. (2012). Differential DNA methylation in purified human blood cells: implications for cell lineage and studies on disease susceptibility. *PLoS One* 7:e41361. 10.1371/journal.pone.0041361 22848472 PMC3405143

[B90] Renal U. S. Data System. (2018). *2018 USRDS Annual Data Report: Epidemiology of Kidney Disease in the United States.* Amsterdam: Elsevier.

[B91] Ritchie M. E. Phipson B. Wu D. Hu Y. Law C. W. Shi W. (2015). limma powers differential expression analyses for RNA-sequencing and microarray studies. *Nucleic Acids Res.* 43:e47. 10.1093/nar/gkv007 25605792 PMC4402510

[B92] Rossing P. Tarnow L. Nielsen F. S. Hansen B. V. Brenner B. M. Parving H. H. (1995). Low birth weight. a risk factor for development of diabetic nephropathy?. *Diabetes* 44 1405–1407. 10.2337/diabetes.44.12.1405 7589846

[B93] Salem R. M. Todd J. N. Sandholm N. Cole J. B. Chen W. M. Andrews D. (2019). Genome-Wide association study of diabetic kidney disease highlights biology involved in glomerular basement membrane collagen. *J. Am. Soc. Nephrol.* 30 2000–2016. 31537649 10.1681/ASN.2019030218 PMC6779358

[B94] Sandholm N. Forsblom C. Makinen V. P. McKnight A. J. Osterholm A. M. He B. (2014). Genome-wide association study of urinary albumin excretion rate in patients with type 1 diabetes. *Diabetologia* 57 1143–1153. 24595857 10.1007/s00125-014-3202-3

[B95] Sandholm N. McKnight A. J. Salem R. M. Brennan E. P. Forsblom C. Harjutsalo V. (2013). Chromosome 2q31.1 associates with ESRD in women with type 1 diabetes. *J. Am. Soc. Nephrol.* 24 1537–1543. 10.1681/asn.2012111122 24029427 PMC3785274

[B96] Sandholm N. Salem R. M. McKnight A. J. Brennan E. P. Forsblom C. Isakova T. (2012). New susceptibility loci associated with kidney disease in type 1 diabetes. *PLoS Genet.* 8:e1002921. 10.1371/journal.pgen.1002921 23028342 PMC3447939

[B97] Sandholm N. Van Zuydam N. Ahlqvist E. Juliusdottir T. Deshmukh H. A. Rayner N. W. (2017). The Genetic Landscape of Renal Complications in Type 1 Diabetes. *J. Am. Soc. Nephrol.* 28 557–574. 27647854 10.1681/ASN.2016020231 PMC5280020

[B98] Sandoval J. Heyn H. Moran S. Serra-Musach J. Pujana M. A. Bibikova M. (2011). Validation of a DNA methylation microarray for 450,000 CpG sites in the human genome. *Epigenetics* 6 692–702. 10.4161/epi.6.6.16196 21593595

[B99] Sapienza C. Lee J. Powell J. Erinle O. Yafai F. Reichert J. (2011). DNA methylation profiling identifies epigenetic differences between diabetes patients with ESRD and diabetes patients without nephropathy. *Epigenetics* 6 20–28. 10.4161/epi.6.1.13362 21150313

[B100] Sarwal M. Chua M. S. Kambham N. Hsieh S. C. Satterwhite T. Masek M. (2003). Molecular heterogeneity in acute renal allograft rejection identified by DNA microarray profiling. *N. Engl. J. Med.* 349 125–138. 10.1056/nejmoa035588 12853585

[B101] Smyth L. J. Duffy S. Maxwell A. P. McKnight A. J. (2014a). Genetic and epigenetic factors influencing chronic kidney disease. *Am. J. Physiol. Renal. Physiol.* 307 F757–F776. 25080522 10.1152/ajprenal.00306.2014

[B102] Smyth L. J. McKay G. J. Maxwell A. P. McKnight A. J. (2014b). DNA hypermethylation and DNA hypomethylation is present at different loci in chronic kidney disease. *Epigenetics* 9 366–376. 10.4161/epi.27161 24253112 PMC4053455

[B103] Smyth L. J. Maxwell A. P. Benson K. A. Kilner J. McKay G. J. McKnight A. J. (2018). Validation of differentially methylated microRNAs identified from an epigenome-wide association study; sanger and next generation sequencing approaches. *BMC Res. Notes* 11:767. 10.1186/s13104-018-3872-x 30373632 PMC6206874

[B104] Stefan M. Zhang W. Concepcion E. Yi Z. Tomer Y. (2014). DNA methylation profiles in type 1 diabetes twins point to strong epigenetic effects on etiology. *J. Autoimmun.* 50 33–37. 10.1016/j.jaut.2013.10.001 24210274 PMC3995844

[B105] Sticht C. Hofele C. Flechtenmacher C. Bosch F. X. Freier K. Lichter P. (2005). Amplification of Cyclin L1 is associated with lymph node metastases in head and neck squamous cell carcinoma (HNSCC). *Br. J. Cancer* 92 770–774. 10.1038/sj.bjc.6602400 15700036 PMC2361871

[B106] Swan E. J. Maxwell A. P. McKnight A. J. (2015). Distinct methylation patterns in genes that affect mitochondrial function are associated with kidney disease in blood-derived DNA from individuals with Type 1 diabetes. *Diabet. Med.* 32 1110–1115. 10.1111/dme.12775 25850930

[B107] Tannukit S. Wen X. Wang H. Paine M. L. (2008). TFIP11, CCNL1 and EWSR1 Protein-protein Interactions, and Their Nuclear Localization. *Int. J. Mol. Sci.* 9 1504–1514. 10.3390/ijms9081504 19122807 PMC2605624

[B108] UK Renal Registry (2019). *UK Renal Registry 21st Annual Report – Data to 31/12/2017*, Bristol, UK. Available online at: https://www.renalreg.org/publications-reports/

[B109] van Zuydam N. R. Ahlqvist E. Sandholm N. Deshmukh H. Rayner N. W. Abdalla M. (2018). A genome-wide association study of diabetic kidney disease in subjects with Type 2 diabetes. *Diabetes* 67 1414–1427. 29703844 10.2337/db17-0914 PMC6014557

[B110] Walaszczyk E. Luijten M. Spijkerman A. M. W. Bonder M. J. Lutgers H. L. (2018). DNA methylation markers associated with type 2 diabetes, fasting glucose and HbA1c levels: a systematic review and replication in a case-control sample of the lifelines study. *Diabetologia* 61 354–368. 10.1007/s00125-017-4497-7 29164275 PMC6448925

[B111] Wettenhall J. M. Smyth G. K. (2004). limmaGUI: a graphical user interface for linear modeling of microarray data. *Bioinformatics* 20 3705–3706. 10.1093/bioinformatics/bth449 15297296

[B112] Woroniecka K. I. Park A. S. Mohtat D. Thomas D. B. Pullman J. M. Susztak K. (2011). Transcriptome analysis of human diabetic kidney disease. *Diabetes* 60 2354–2369. 10.2337/db10-1181 21752957 PMC3161334

[B113] Yaghootkar H. Freathy R. M. (2012). Genetic origins of low birth weight. *Curr. Opin. Clin. Nutr. Metab. Care* 15 258–264. 10.1097/mco.0b013e328351f543 22406741

[B114] Zhou F. Wang R. Yuan P. Ren Y. Mao Y. Li R. (2019). Reconstituting the transcriptome and DNA methylome landscapes of human implantation. *Nature* 572 660–664. 10.1038/s41586-019-1500-0 31435013

[B115] Zhou T. He X. Cheng R. Zhang B. Zhang R. R. Chen Y. (2012). Implication of dysregulation of the canonical wingless-type MMTV integration site (WNT) pathway in diabetic nephropathy. *Diabetologia* 55 255–266. 10.1007/s00125-011-2314-2 22016045

[B116] Zhou W. Laird W. Shen H. (2017). Comprehensive characterization, annotation and innovative use of Infinium DNA methylation BeadChip probes. *Nucleic Acids Res.* 45:e22. 10.1093/nar/gkw967 PMC5389466 27924034

