# Supplementary material for: DNA Methylation Associated With Diabetic Kidney Disease in Blood-Derived DNA
Source: Front Cell Dev Biol. 2020 Oct 15;8:561907. doi: 10.3389/fcell.2020.561907 (PMC7593403; doi:10.3389/fcell.2020.561907)
Supplement: Supplementary file 1 [file Data_Sheet_1.pdf]

frontiers

Online Supplementary Material

Supplementary Table 1: Comparison of the average and standard deviation of proportional white cell counts between cases and controls.

	CD8+ T		CD4+ T		natural killer cells		B cells		monocytes		granulocytes	
	**Average**	**SD**	**Average**	**SD**	**Average**	**SD**	**Average**	**SD**	**Average**	**SD**	**Average**	**SD**
**Cases**	0.052	0.039	0.194	0.086	0.079	0.047	0.048	0.036	0.074	0.029	0.572	0.137
**Controls**	0.049	0.040	0.189	0.078	0.069	0.044	0.047	0.050	0.071	0.030	0.593	0.135
**t test**	0.66		0.69		0.12		0.81		0.48		0.24	

Supplementary Table 2: DAVID annotation of top-ranked genes from the 450K discovery analysis.

**CASS4**	Cas scaffolding protein family member 4
PUBMED_ID	11780052, 12477932, 14702039, 15489334, 18083107, 18088087, 18256281
**CCNL1**	cyclin L1
PUBMED_ID	11042152, 11683997, 11980906, 12414649, 12477932, 12975309, 14623875, 14702039, 15302935, 15489334, 15700036, 16598186, 17494991, 18187866, 18216018, 18220336, 18669648
**CNOT6L**	CCR4-NOT transcription complex, subunit 6-like
KEGG_PATHWAY	RNA degradation
PUBMED_ID	14702039, 15815621, 16344560, 17353931, 17452450, 17974005, 18377426
**DDX5**	DEAD (Asp-Glu-Ala-Asp) box polypeptide 5
GENETIC_ASSOCIATION_DB_DISEASE	hepatitis C, chronic
KEGG_PATHWAY	Spliceosome
PUBMED_ID	10409727, 10648785, 10837141, 11250900, 11279182, 11790298, 11991638, 12101238, 12477932, 12527917, 12595555, 12665590, 12738788, 14702039, 15113910, 15298701, 15304501, 15464984, 15489334, 15592455, 15635413, 15660129, 15782174, 15927448, 15950181, 16344560, 16697732, 17018282, 17220478, 17287340, 17353931, 17369852, 17384675, 17540040, 17699760, 17724023, 17924679, 18005418, 18083107, 18239468, 18377426, 18669648, 18691976, 18794152, 18829551, 18954305, 19171422, 19224332, 1996094, 2349099, 2451786, 2762324, 7525583, 7774924
**DLX5**	distal-less homeobox 5
PUBMED_ID	10451362, 10516593, 11084035, 11675124, 11959851, 12145306, 12477932, 12690205, 12782124, 12853948, 14702039, 15489334, 15954098, 16344560, 16467978, 16582099, 17363207, 17701895, 18029348, 18316591, 18413826, 7907794, 7987313, 8889549, 9111364
**DST**	dystonin

PUBMED_ID	10428034, 10637308, 10727295, 11323216, 11375975, 11751855, 12168954, 12421765, 12477932, 12482924, 12542537, 12802069, 14574404, 14576348, 14581450, 14702039, 14705806, 15231748, 15342556, 15489334, 15560761, 15725571, 16344560, 16512878, 17043677, 17081983, 1712022, 17161423, 1717441, 17287340, 17920818, 17924679, 18187866, 18207369, 18669648, 18781797, 2045679, 2090522, 2276744, 2461961, 2579167, 2645368, 3880796, 7818282, 7919656, 8010969, 8345227, 8349819, 8575775, 8621649, 8889548, 8889549, 9872452
**ELF1**	E74-like factor 1 (ets domain transcription factor)
PUBMED_ID	10207087, 10377039, 10976766, 11210123, 11884456, 12477932, 12727645, 14702039, 14970218, 15057823, 1527846, 15302935, 1545787, 15489334, 15566516, 16464244, 16964243, 17525332, 17652178, 18029348, 18220336, 18314487, 18378679, 18669648, 18714041, 7862168, 8493578, 8756667, 9094628, 9524226, 9668064
**HSPBAP1**	HSPB (heat shock 27kDa) associated protein 1
PUBMED_ID	10751411, 11978969, 12477932, 12939738, 14702039, 15489334, 16169070, 17568411
**IL27RA**	interleukin 27 receptor, alpha
PUBMED_ID	11057672, 12121660, 12477932, 12734330, 12975309, 14764690, 15489334, 17703412, 18003935, 19258923, 9600072
**KIAA1586**	KIAA1586
PUBMED_ID	10997877, 12477932, 14574404, 14702039, 15489334
**PIK3CG**	phosphoinositide-3-kinase, catalytic, gamma polypeptide
GENETIC_ASSOCIATION_DB_DISEASE	longevity
KEGG_PATHWAY	Inositol phosphate metabolism, ErbB signaling pathway, Chemokine signaling pathway, Phosphatidylinositol signaling system, mTOR signaling pathway, Apoptosis, VEGF signaling pathway, Focal adhesion, Toll-like receptor signaling pathway, Jak-STAT signaling pathway, Natural killer cell mediated cytotoxicity, T cell receptor signaling pathway, B cell receptor signaling pathway, Fc epsilon RI signaling pathway, Fc gamma R-mediated phagocytosis, Leukocyte transendothelial migration, Neurotrophin signaling pathway, Regulation of actin cytoskeleton, Insulin signaling pathway, Progesterone-mediated oocyte maturation, Type II diabetes mellitus, Aldosterone-regulated sodium reabsorption, Pathways in cancer, Colorectal cancer, Renal cell carcinoma, Pancreatic cancer, Endometrial cancer,

	Glioma, Prostate cancer, Melanoma, Chronic myeloid leukemia, Acute myeloid leukemia, Small cell lung cancer, Non-small cell lung cancer,
PUBMED_ID	10318860, 10358071, 10386953, 10488142, 10542052, 10571082, 10579793, 10580505, 10816567, 10972292, 11090628, 11136978, 11154208, 11156964, 11181995, 11238738, 11259422, 11416136, 11577104, 11756194, 11756422, 11861293, 11994280, 12077252, 12115604, 12163475, 12242282, 12435806, 12464013, 12473596, 12477932, 12502714, 12507995, 12529294, 12545160, 12551992, 12606772, 12690205, 12757856, 12774123, 12853948, 13130092, 14583609, 14602571, 14605879, 14622970, 15001544, 15044087, 15046613, 15326564, 15489334, 15496972, 15543611, 15558024, 15582274, 15611065, 15668028, 15678501, 15733066, 15741161, 15797027, 15845472, 15878979, 15901830, 15932879, 15993382, 16081599, 16094730, 16127437, 16204373, 16219695, 16286246, 16291747, 16325767, 16328013, 16430866, 16432180, 16507111, 16527821, 16533525, 16647110, 16772787, 16943418, 17001038, 17008323, 17045571, 17126402, 17158252, 17173040, 17235455, 17286201, 17290298, 17302605, 17303701, 17375124, 17410437, 17459875, 17497700, 17515959, 17551921, 17646933, 17658464, 17681345, 17716861, 17724079, 17855501, 17868340, 17878755, 17900864, 17901375, 17942603, 17971428, 17998459, 18025271, 18060476, 18079201, 18187452, 18201823, 18245521, 18273051, 18299320, 18338817, 18374639, 18395956, 18434090, 18451027, 18451140, 18458059, 18467332, 18469852, 18484710, 18495460, 18499442, 18502332, 18511807, 18515365, 18520805, 18535744, 18546844, 18552129, 18558630, 18587247, 18593948, 18603327, 18615538, 18625725, 18632611, 18644721, 18652687, 18663085, 18675921, 18678286, 18711316, 18720410, 18722093, 18725974, 18728784, 18754654, 18766172, 18769058, 18771702, 18810319, 18824170, 18844239, 18849971, 18945831, 18973553, 18983267, 18996102, 19011679, 19017490, 19058789, 19059380, 19079138, 19086053, 19103290, 19152965, 19179443, 19211763, 19224914, 19261616, 19269083, 7520444, 7606002, 7624799, 8313897, 8798481, 9341793, 9394803, 9446654, 9446795, 9507010, 9708406, 9747873, 9808187
**RAD50**	RAD50 homolog (S. cerevisiae)
GENETIC_ASSOCIATION_DB_DISEASE	breast cancer, lymphoma, Non-Hodgkin's,
KEGG_PATHWAY	Homologous recombination, Non-homologous end-joining,
OMIM_DISEASE	Genome-Wide Scan on Total Serum IgE Levels Identifies FCER1A as Novel Susceptibility Locus,

PUBMED_ID	10346816, 10415333, 10426999, 10783165, 10802669, 10839544, 10888888, 11096100, 11238951, 11438675, 11533665, 11741547, 11790298, 11809878, 11877377, 12007281, 12124628, 12152085, 12384589, 12477932, 12607003, 12607005, 12660252, 12805565, 14550546, 14684699, 14698290, 15064416, 15100233, 15180989, 15234984, 15279769, 15302935, 15456891, 15489334, 15635413, 15653682, 15723659, 15790808, 15937485, 16051665, 16087684, 16163361, 16254336, 16385572, 16474176, 16788144, 16905549, 17081983, 17169801, 17349953, 17426050, 17456004, 17524422, 17525332, 17526493, 17534377, 17567994, 17693401, 17694070, 17700070, 17715134, 17898048, 17932350, 18219098, 18469862, 18596698, 18632984, 18638378, 18669648, 18829552, 18830935, 18846228, 18950845, 19064565, 19064572, 19124506, 19131452, 19190165, 19197236, 19244322, 19383352, 8756642, 9315668, 9590181, 9651580, 9705271
**RFC3**	replication factor C (activator 1) 3, 38kDa
KEGG_PATHWAY	DNA replication, Nucleotide excision repair, Mismatch repair
PUBMED_ID	10051561, 10884395, 11572977, 11907025, 12171929, 12192049, 12477932, 12766176, 12930902, 14605214, 14702039, 15489334, 15635413, 16079077, 16344560, 17456004, 17525332, 19237606, 7774928, 8125298, 8441605, 8692848, 9092549, 9111189, 9121429, 9228079, 9373149, 9488738, 9822671
**RUFY1**	RUN and FYVE domain containing 1
KEGG_PATHWAY	Endocytosis
PUBMED_ID	11172003, 11877430, 12477932, 14617813, 14702039, 15342556, 15489334, 16344560, 16959974, 17525332,
**TBC1D1**	TBC1 (tre-2/USP6, BUB2, cdc16) domain family, member 1
PUBMED_ID	10470851, 10965142, 12168954, 12477932, 14702039, 15231748, 15324660, 15489334, 15592455, 16303743, 16893906, 17274760, 18325908, 19077034, 19115052
**ZNF187 (ZSCAN26)**	zinc finger protein 187
PUBMED_ID	12477932, 14574404, 14702039, 15489334, 1569959, 16344560, 17974005
**ISPD (CRPPA)**	notch1-induced protein
PUBMED_ID	10737800, 11181995, 12690205, 12853948, 9847074

Supplementary Table 3: Most associated SNPs, based on genome-wide association study with significance values adjusted for genomic control and clinical covariates, in the region of interest from the 450K discovery analysis.

Locus	Δβ<br>DN vs DC	*P*<br>DN vs DC	Most associated SNP	Pgc for SNP
**cg10905876**	0.22	8.90E-22	rs16888186	7.9E-04
**cg20419181**	0.26	5.20E-23	rs2114014	1.18E-03
**cg03037561**	0.2	3.20E-18	rs2189735	4.56E-03
**cg25418748**	0.21	5.00E-22	rs11541393	8.82E-03
**cg15189015**	0.22	3.00E-22	rs6501732	1.12E-02
**cg10463299**	0.23	2.30E-22	rs17462136	1.27E-02
**cg16595484**	0.22	2.40E-21	rs17212608	1.34E-02
**cg04861640**	0.23	3.70E-24	rs1778508	3.81E-02
**cg02339392**	0.21	2.20E-22	rs1778508	3.81E-02
**cg07979357**	0.2	1.80E-22	rs2420413	0.079
**cg21739208**	0.22	6.80E-23	rs7768651	0.12
**cg21935083**	0.22	5.40E-23	rs17772583	0.12
**cg00618312**	0.2	2.40E-22	rs1056835	0.13
**cg21829265**	0.21	4.10E-23	rs9367704	0.18
**cg16203607**	0.21	2.10E-23	rs8071264	0.19

**cg11671265**	0.24	5.00E-18	rs17448536	0.23
**cg24115040**	0.22	2.10E-22	rs3757492	0.25
**cg01667324**	0.2	5.30E-21	rs3135527	0.29
**cg26399113**	0.22	1.00E-12	rs4719110	0.34
**cg08779777**	0.26	1.50E-20	rs3173908	0.36
**cg07242860**	0.22	7.20E-23	rs7722108	0.41
**cg02399570**	0.21	8.60E-22	rs9878138	0.49

Supplementary Figure 1: Gene expression evidence generated in and derived from NephroSeq for *CCNL1 & ZNF187* genes. (a) Distribution of *CCNL1* and (b) *ZNF187* mRNA in kidney tissues. (c) Summary of significant analyses returned from NephroSeq. (d) Expression of *CCNL1* based on analysis of kidney and blood-derived RNA. (e) Expression of ZNF187 based on analysis of kidney and blood-derived RNA

(a)

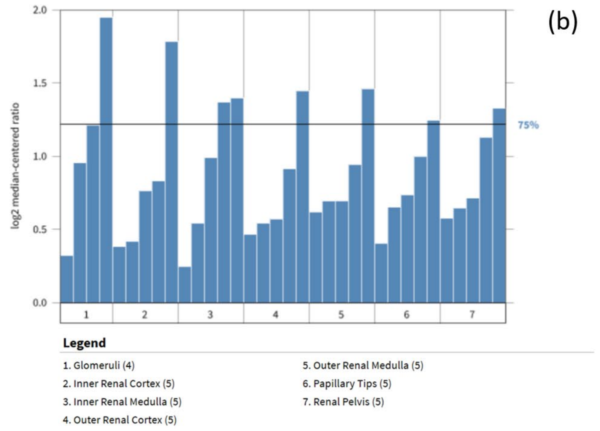

(b)

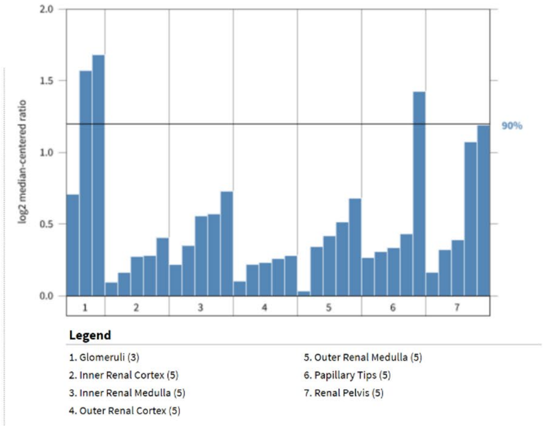

(c)

Analyses for CCNL1

Summary | Details — Threshold by: p VALUE 0.05, r VALUE 0.5, FOLD CHANGE 1.5

ANALYSIS TYPE	SIGNIFICANT* ANALYSES	TOTAL UNIQUE ANALYSES
Tissue Type Analyses	9	40
GFR Analyses	5	41
Age Analyses	4	44
Blood Pressure Analyses	3	23
Disease vs. Control Analyses	3	50
Weight Analyses	3	34
Race Ethnicity Analyses	2	46
Serum Creatinine Analyses	2	45
Transplant Analyses	2	9
Body Mass Index Analyses	1	25
Fasting Blood Glucose Analyses	1	4
Proteinuria Analyses	1	53
Sex Analyses	1	49

Analyses for ZNF187

Summary | Details — Threshold by: p VALUE 0.05, r VALUE 0.5, FOLD CHANGE 1.5

ANALYSIS TYPE	SIGNIFICANT* ANALYSES	TOTAL UNIQUE ANALYSES
Serum Creatinine Analyses	5	46
Tissue Type Analyses	5	39
GFR Analyses	4	31
Proteinuria Analyses	3	42
Transplant Analyses	2	14
Weight Analyses	2	33
Blood Pressure Analyses	1	23
Race Ethnicity Analyses	1	36

(d)

ANALYSIS	p VAL	r VAL	FOLD CHANGE	DATASET
Disease vs. Control Analysis<br>Thin Basement Membrane Disease vs. Healthy Living Donor	3.55e-9		-1.693	Ju CKD Glom
Disease vs. Control Analysis<br>Chronic Kidney Disease vs. Normal Kidney (Discovery Set)	1.84e-5		-1.550	Nakagawa CKD Kidney

ANALYSIS	p VAL	r VAL	FOLD CHANGE	DATASET
Transplant Analysis<br>Acute Rejection vs. No Rejection (Males)	0.011		1.751	Gunther Transplant Blood
Transplant Analysis<br>Acute Rejection vs. No Rejection (All Measured Samples)	0.012		1.597	Gunther Transplant Blood
GFR (MDRD) Analysis<br>(IgA Nephropathy Samples)	0.022	0.783		Cox IgAN Blood

(e)

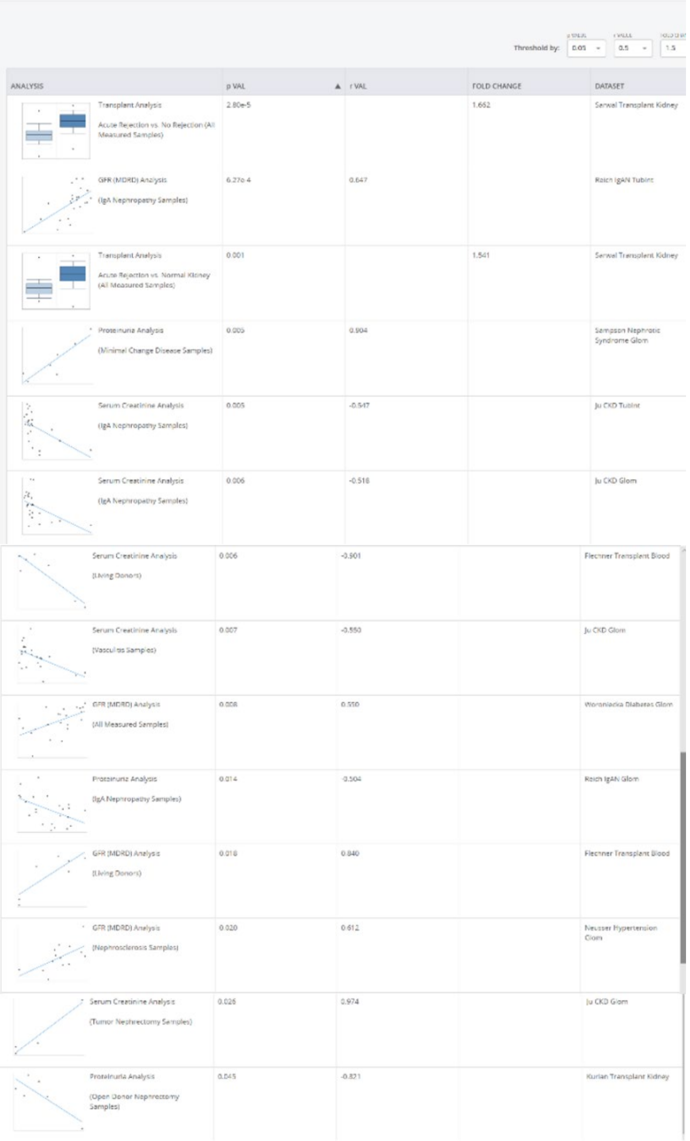

Threshold by: p VALUE 0.05 | r VALUE 0.5 | FOLD CHANGE 1.5

ANALYSIS	p VAL	r VAL	FOLD CHANGE	DATASET
Transplant Analysis<br>Acute Rejection vs. No Rejection (All Measured Samples)	2.80e-5		1.662	Sarwal Transplant Kidney
GFR (MDRD) Analysis<br>(IgA Nephropathy Samples)	6.27e-4	0.647		Reich IgAN Tubint
Transplant Analysis<br>Acute Rejection vs. Normal Kidney (All Measured Samples)	0.001		1.541	Sarwal Transplant Kidney
Proteinuria Analysis<br>(Minimal Change Disease Samples)	0.005	0.904		Sampson Nephrotic Syndrome Glom
Serum Creatinine Analysis<br>(IgA Nephropathy Samples)	0.005	-0.547		Ju CKD Tubint
Serum Creatinine Analysis<br>(IgA Nephropathy Samples)	0.006	-0.518		Ju CKD Glom
Serum Creatinine Analysis<br>(Living Donors)	0.006	-0.901		Flechner Transplant Blood
Serum Creatinine Analysis<br>(Vasculitis Samples)	0.007	-0.550		Ju CKD Glom
GFR (MDRD) Analysis<br>(All Measured Samples)	0.008	0.550		Woroniecka Diabetes Glom
Proteinuria Analysis<br>(IgA Nephropathy Samples)	0.014	-0.504		Reich IgAN Glom
GFR (MDRD) Analysis<br>(Living Donors)	0.018	0.840		Flechner Transplant Blood
GFR (MDRD) Analysis<br>(Nephrosclerosis Samples)	0.020	0.612		Neusser Hypertension Glom
Serum Creatinine Analysis<br>(Tumor Nephrectomy Samples)	0.026	0.974		Ju CKD Glom
Proteinuria Analysis<br>(Open Donor Nephrectomy Samples)	0.045	-0.821		Kurian Transplant Kidney
